# Effect of Different Host Plants on the Diversity of Gut Bacterial Communities of *Spodoptera frugiperda* (J. E. Smith, 1797)

**DOI:** 10.3390/insects14030264

**Published:** 2023-03-08

**Authors:** Shipeng Han, Yayuan Zhou, Da Wang, Qiuju Qin, Peng Song, Yunzhuan He

**Affiliations:** College of Plant Protection, Hebei Agricultural University, Baoding 071000, China

**Keywords:** *Spodoptera frugiperda*, fall armyworm, 16S rRNA, gut microbiota, host plants

## Abstract

**Simple Summary:**

The gut microbes are important to the insect physiology and behavior. *Spodoptera frugiperda* (J. E. Smith) is a worldwide migratory invasive and polyphagous pest, which causes great damage to various host crops in China. The structures of gut bacterial community of *S. frugiperda* feeding on different host plants are largely unexplored. In this study, high-throughput sequencing was used to compare the gut bacterial communities of *S. frugiperda* reared on different host plants. The results confirmed that the host plants can significantly affect the gut bacterial diversity and structure of *S. frugiperda*. This study lays a foundation for further studies on the function of gut bacteria of *S. frugiperda* and its adaptive mechanism to the host.

**Abstract:**

Intestinal symbiotic bacteria have formed an interdependent symbiotic relationship with many insect species after long-term coevolution, which plays a critical role in host growth and adaptation. *Spodoptera frugiperda* (J. E. Smith) is a worldwide significant migratory invasive pest. As a polyphagous pest, *S. frugiperda* can harm more than 350 plants and poses a severe threat to food security and agricultural production. In this study, 16S rRNA high-throughput sequencing technology was used to analyze the diversity and structure of the gut bacteria of this pest feeding on six diets (maize, wheat, rice, honeysuckle flowers, honeysuckle leaves, and Chinese yam). The results showed that the *S. frugiperda* fed on rice had the highest bacterial richness and diversity, whereas the larvae fed on honeysuckle flowers had the lowest abundance and diversity of gut bacterial communities. Firmicutes, Actinobacteriota, and Proteobacteria were the most dominant bacterial phyla. PICRUSt2 analysis indicated that most of the functional prediction categories were concentrated in metabolic bacteria. Our results confirmed that the gut bacterial diversity and community composition of *S. frugiperda* were affected significantly by host diets. This study provided a theoretical basis for clarifying the host adaptation mechanism of *S. frugiperda*, which also provided a new direction to improve polyphagous pest management strategies.

## 1. Introduction

Most insect guts contain a multitude of commensal microorganisms, which form an interdependent symbiotic relationship with insects after long-term coevolution [1,2]. With advances in high-throughput sequencing technology, the focus of research has gradually transferred to the composition and functions of microorganisms related to insects. Bacteria are the dominant group in most insect microbial communities and contribute to various insect life processes, such as digestion and absorption of nutrients, immune defense response, degrade exogenous biological toxins, pesticide resistance, reproductive regulation, and host adaptability regulation [3,4,5,6,7,8,9,10,11].

Previous studies indicated that the composition of the gut microbiota communities of insects is affected by multiple endogenous and exogenous factors. Firstly, different species and developmental stages of insect hosts had distinctive intestinal microbial compositions [12,13,14]. Liu et al. reported that the structures of gut microbes of four fruit borers showed significant differences [15]. Gao et al. found that Firmicutes were the main bacterial communities in 3 L and 5 L of *Spodoptera exigua*, and that Proteobacteria dominated the other life stages [16]. Secondly, the bacterial composition of insect hosts was significantly affected by their geographical distribution. The bacterial structures between diverse sampling sites of corn earworm were significantly different [17]. Thirdly, some exogenous factors could also change the intestinal microbiota communities, such as temperature, pesticide, and diet [10,15,18,19]. Among them, diet is the main external factor. After continuous rearing with peas, the diversity of gut microbes in *Plutella xylostella* was significantly decreased [20]. In addition, the host plants strongly affected the structure and abundance of microorganisms in *Grapholita molesta*, as reported by Yuan et al. [21]. Kim et al. reported similar results in *Monochamus alternatus* reared with host tree logs and artificial diets [22].

*Spodoptera frugiperda* is a global invasive pest native to tropical and subtropical Americas [23]. At present, *S. frugiperda* has invaded 46 countries or regions of Africa and nine countries of Asia facilitated by its strong migration capability [24,25]. This pest has already invaded 29 provinces in China, and in 2019, the damage of *S. frugiperda* was confirmed for the first time in Yunnan Province [26]. As a notorious polyphagous pest, *S. frugiperda* can feed on more than 350 plant species belonging to 76 plant families, and maize is one of the main host plants of *S. frugiperda* [27]. Under laboratory conditions, *S. frugiperda* could complete the generation cycle on many common crops, such as cotton, castor, cauliflower, oilseed rape, soybean, and sunflower [28,29]. In addition, our previous studies found that *S. frugiperda* could complete life cycles on *Lonicera japonica* Thunb and *Dioscorea oppositifolia* Turczaninow, two traditional Chinese medicines cultivated in China [30]. Therefore, *S. frugiperda* poses a serious threat to the agricultural production and security of China.

Lepidoptera contains a large number of agricultural pests. In recent years, more and more research on gut microbial communities of Lepidoptera insects have been reported, such as *Bombyx mori*, *S. exigua*, *Spodoptera litura*, and several fruit borers [12,15,16,31]. Similar to other Lepidopteran, the gut bacterial community of *S. frugiperda* is also significantly affected by host plants. Mason et al. found that the gut bacterial abundance of *S. frugiperda* increased when larvae were fed with gamma-irradiated corn leaves, and decreased when fed an artificial diet [32]. Lv et al. reported that the microbial diversity on maize was higher than that on wild oat and pepper [33]. However, there is no research on the effect of gut bacterial communities in *S. frugiperda* feeding on Chinese traditional medicine crops. In this study, we compared gut bacterial communities of *S. frugiperda* reared on different host plants (three major food crops: maize, wheat, and rice, and two Chinese herbal medicines: honeysuckle and Chinese yam). Our findings enable further understanding of the effects of host plants on gut microbiota communities of *S. frugiperda* and lay a foundation for revealing the host adaptability of this pest.

## 2. Materials and Methods

### 2.1. Insect Rearing and Sample Collection

*Spodoptera frugiperda* was obtained from the Henan Academy of Agricultural Sciences (Zhengzhou, China) and reared on maize seedlings for more than 3 years at 26 ± 1 °C, 60–70% relative humidity, and 16 L:8D photoperiod in the artificial climate incubator.

Newly hatched larvae were reared with maize (*Zea mays* L. Zhengdan 958), wheat (*Triticum aestivum* L. Jimai 22), rice (*Oryza sativa* L. Zhenghan 10), the flower of honeysuckle (*Lonicera japonica* Thunb. Juhua 1), the leaves of honeysuckle (*Lonicera japonica* Thunb. Juhua 1) and Chinese yam (*Dioscorea oppositifolia* Turczaninow Xiaobaizui) until fifth instar. Then, 70% ethanol was used to sterilize the larvae for 1 min followed by three sterile water washes. Under a stereoscope, the midgut was dissected in sterile phosphate buffered saline. Larvae fed each different diet were obtained from three different batches and each batch contained 15 to 20 individuals.

### 2.2. DNA Extraction, Library Preparation, and High-Throughput Sequencing

The genomic DNA was extracted from all samples using DNeasy^®^ PowerSoil^®^ Pro Kit (QIAGEN, Dusseldorf, Germany), according to the instruction. DNA quality and quantity were detected with a NanoDrop spectrophotometer (Thermo Fisher Scientific, Waltham, MA, USA) and the integrity was evaluated using 1% agarose gel. The total DNA was stored at −80 °C. The universal primer pair 338F and 806R was used to amplify the hypervariable regions V3–V4 of the bacterial 16S rRNA gene. The PCR products were detected and purified on 2% agarose gels. The recovered products were quantified using Quantus™ Fluorometer (Promega, Madison, WI, USA) and homogenized to form a sequencing library. Sequencing was conducted on Illumina Miseq PE300 (Illumina, San Diego, CA, USA). Raw reads were submitted to the National Center for Biotechnology Information (NCBI) Sequence Read Archive (SRA) database (accession No. PRJNA869195).

### 2.3. Statistical and Bioinformatics Analysis

FLASH (version 1.2.7) was used to truncate the barcode and primer sequences and reassign the paired-end reads, the criteria of overlap was greater than 10 bp and a had mismatch rate of less than 0.02 [34,35]. Then, the high-quality sequences were classified into multiple operational taxonomic units (OTUs) based on 97% similarity truncation value with UPARSE (version 7.1) [36]. The representative sequences from each OTU were screened and annotated against the 16S rRNA database (Silva database version 138) by RDP classifier (version 2.2) with a confidence threshold at 70% [37]. OTU abundance information was normalized by the smallest sequence for subsequent analyses. Alpha diversity analysis was executed by Mothur (version 1.30.2) software to evaluate the abundance and diversity of microbial communities [38]. We used QIIME (version 1.9.1) to conduct principal coordinate analysis (PCoA) based on the Bray–Curtis similarities index, which was usually applied to reveal the differences in microbial community structures among different treatment groups [39]. To intuitively show the composition and differences of communities, the columnar cumulative diagram and heat map were generated using R vegan package [15]. Linear discriminant analysis (LDA) effect size (LEfSe) was used to identify the species with statistical differences in an abundance of microbial communities. PICRUSt2 was used to annotate pathways of OTUs against the KEGG databases to predict the abundance of microbiota functions [40,41]. All statistical analyses were performed by SPSS (version 22.0) with a one-way ANOVA analysis followed by Tukey’s test (α = 0.05).

## 3. Results

### 3.1. General Profile of 16S rRNA Sequencing Data

A total of 960, 641 high-quality reads with an average length of 428 bp were obtained (Table S1). The sample rarefaction curve indicated that the sequencing volume and depth of the samples were saturated (Figure S1). Furthermore, the Good’s coverage reflected sequencing integrity. In this study, the coverage of each sample was above 99%, showing that most species in the samples were detected (Table S2). The high-quality sequences were divided into 431 OTUs by cluster analysis, including 21 phyla, 43 classes, 101 orders, 167 families, 292 genera, and 380 species.

### 3.2. Comparison of the Bacterial Communities

**Figure 1:**
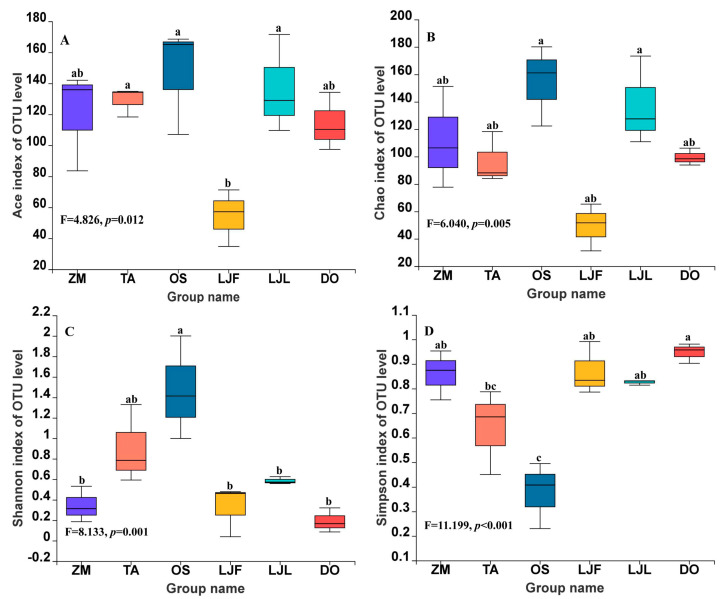
Box plots of gut microbiota diversity of *Spodoptera frugiperda* fed on different host plants. (**A**) ACE index, (**B**) Chao index, (**C**) Shannon index, (**D**) Simpson index. Abbreviations in the figure represent different treatments: ZM, maize; TA, wheat; OS, rice; LJF, honeysuckle flowers; LJL, honeysuckle leaves; and DO, Chinese yam. Lowercase letters above the boxes indicate significant differences between different treatments (one−way ANOVA, Tukey’s test, α = 0.05).

The alpha diversity analysis reflected significant differences in the bacterial communities of *S. frugiperda* larvae feeding on six different host plants (Figure 1; Table S2). The Ace and Chao1 indices indicated the abundance of microbial community, and the Shannon and Simpson indices reflected the diversity of microbial community (Figure 1; Table S2). *Spodoptera frugiperda* larvae fed on rice had the highest Ace and Chao abundance values, the highest Shannon diversity values, and the lowest Simpson diversity values. In contrast, the larvae fed on flowers of honeysuckle had the lowest abundance and diversity of gut bacterial communities (Figure 1).

**Figure 2:**
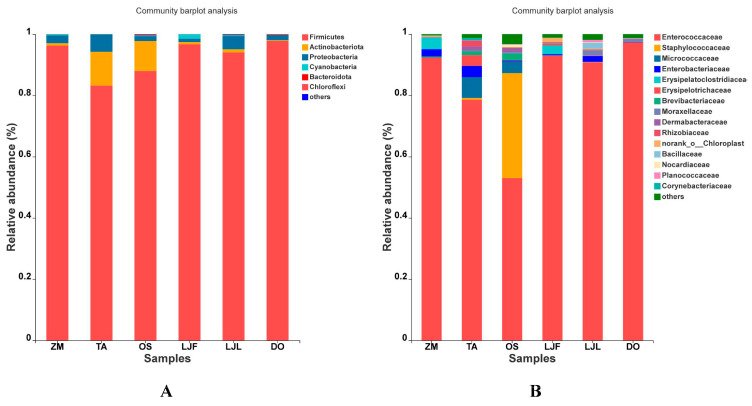
The relative abundance of dominant bacteria in *Spodoptera frugiperda* fed on different hosts. (**A**) phylum levels; (**B**) family levels. Abbreviations in the figure represent different treatments: ZM, maize; TA, wheat; OS, rice; LJF, honeysuckle flowers; LJL, honeysuckle leaves; and DO, Chinese yam.

We identified the gut microbial communities of each treatment at different taxonomic levels. The main phyla of *S. frugiperda* larvae were Firmicutes, Actinobacteriota, Proteobacteria, Cyanobacteria, and Bacteroidota, respectively. Among them, Firmicutes was the main component of gut microbes, the relative abundance accounting from 80.80 ± 12.71% to 98.97 ± 0.44%. Firmicutes were more abundant among *S. frugiperda* larvae fed on maize, honeysuckle flowers and leaves, and Chinese yam than those fed on wheat and rice. The relative abundances of Actinobacteriota in *S. frugiperda* larvae fed on wheat (13.33 ± 3.89%) and rice (11.90 ± 2.26%) were significantly higher than those fed on other host plants. In addition, the relative abundance of Proteobacteria was much higher in *S. frugiperda* larvae fed on honeysuckle leaves (15.17 ± 0.87%) than those fed on honeysuckle flowers (Table 1; Figure 2). At the family level, Enterococcaceae was prevalent in all samples, accounting from 68.50 ± 9.75% to 97.48 ± 2.07%. Relative abundances of Staphylococcaceae in *S. frugiperda* larvae fed on rice (34.31 ± 11.58%) was significantly higher than those fed on other host plants (Table 1; Figure 2).

**Figure 3:**
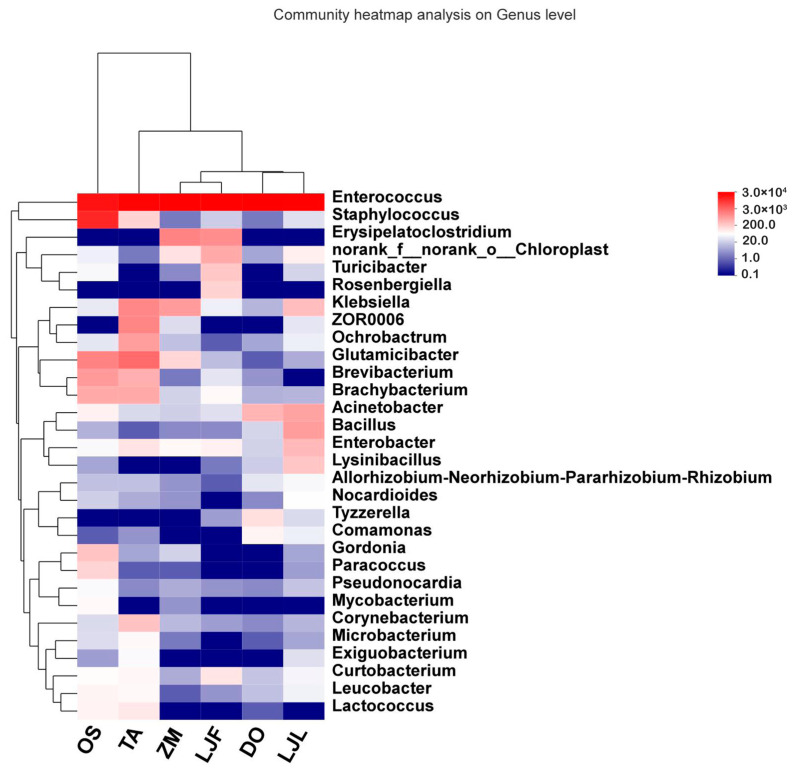
Heatmap of the top 30 most abundant genera in the bacterial community of *Spodoptera frugiperda* fed on different hosts. Abbreviations in the figure represent different treatments: ZM, maize; TA, wheat; OS, rice; LJF, honeysuckle flowers; LJL, honeysuckle leaves; and DO, Chinese yam. The columns represent the samples and rows represent the bacterial at the genus level. The color scale represents the normalized values of relative abundances by log10.

The dynamic patterns in the gut bacterial communities of *S. frugiperda* feeding on different host plants heat map performed with the top 30 abundant genera is shown in Figure 3. The columns represent the samples and the rows represent the bacterial at the genus level. The bacterial communities of *S. frugiperda* larvae feeding on honeysuckle leaves and Chinese yam were converged to the same branch, while that on rice was in the outermost branch. *Enterococcus* was the absolute dominant genus of the gut microbiota in all samples. *Staphylococcus* only had a high abundance of *S. frugiperda* larvae fed on rice.

**Figure 4:**
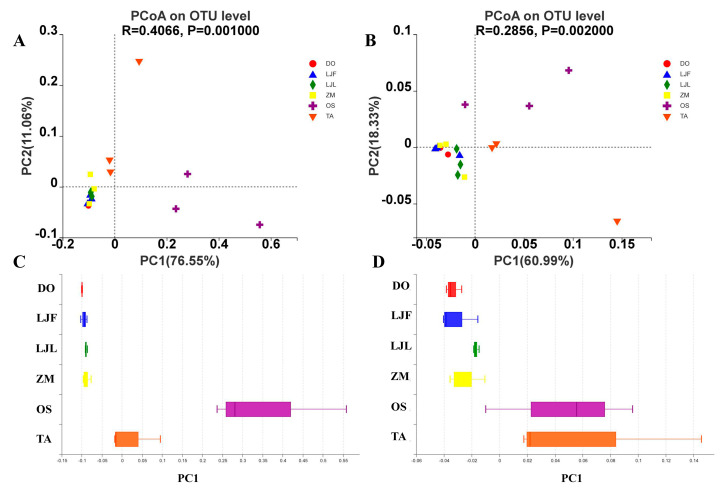
Principal coordinate analysis (PCoA) between different treatments. (**A**,**C**) based on the Bray−Curtis algorithm; (**B**,**D**) based on the weighted Unifrac algorithm. Abbreviations in the figure represent different treatments: ZM, maize; TA, wheat; OS, rice; LJF, honeysuckle flowers; LJL, honeysuckle leaves; and DO, Chinese yam.

The PCoA scatter plot revealed that the principal coordinates represent the two eigenvalues that contribute to the largest variation between the samples (Figure 4). Based on Bray–Curtis algorithm, the principal coordinates explained 76.55% and 11.06% of the data variation, respectively. The ANOSIM analysis showed that the structure of gut bacterial community among different groups was significantly different (R = 0.4066, *p* = 0.001) (Figure 4A,C). According to weighted Unifrac algorithm, the influence degrees of data changes were 60.99% and 18.33%. The ANOSIM analysis also revealed significant differences in the structure of gut microbiota in different samples (R = 0.2856, *p* = 0.002) (Figure 4B,D). The results of both algorithms showed that the bacterial communities of *S. frugiperda* larvae fed on rice and wheat were dispersed with other larvae groups.

**Figure 5:**
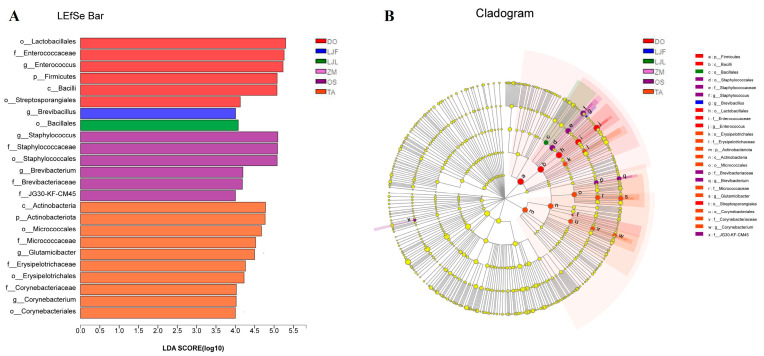
Taxonomic biomarkers of bacterial community in *Spodoptera frugiperda* feeding on different hosts. Scores (**A**) and cladogram (**B**) of taxonomic biomarkers were identified by linear discriminant analysis (LDA) using LEfSe. Lowercase letters represents different bacterial taxa. Different colors represent different treatments, and the different colored nodes represent the bacteria taxa enriched in that treatment. Nodes with yellow indicate no significant difference. Abbreviations in the figure represent different treatments: ZM, maize; TA, wheat; OS, rice; LJF, honeysuckle flowers; LJL, honeysuckle leaves; and DO, Chinese yam.

The LEfSe was used to analyze the main bacterial community of different taxa at various levels (phylum to genus) in different groups (LDA threshold was 4) (Figure 5A). Simultaneously, we drew an evolutionary branching to further understand the difference in bacterial community between different treatments (Figure 5B). As shown in Figure 5, the main gut microbial community of *S. frugiperda* larvae feeding on Chinese yam were Firmicutes and Actinomycetes, including Lactobacillales, *Enterococcus*, Bacilli, and Streptosporangiales. Only one dominant microbial species was detected in the honeysuckle flower-feeding group, which belonged to the *Brevibacillus* genus of Firmicutes. The Bacillales was a dominant taxon of *S. frugiperda* feeding on the leaf of honeysuckle. For rice-feeding group, Staphylococcaceae and Brevibacteriaceae were the main bacterial communities. There were five microbial species of *S. frugiperda* larvae feeding on wheat, four of which were exclusive to Firmicutes and the other belonging to Actinomycetes (Figure 5).

### 3.3. Functional Prediction of Microbiota

**Figure 6:**
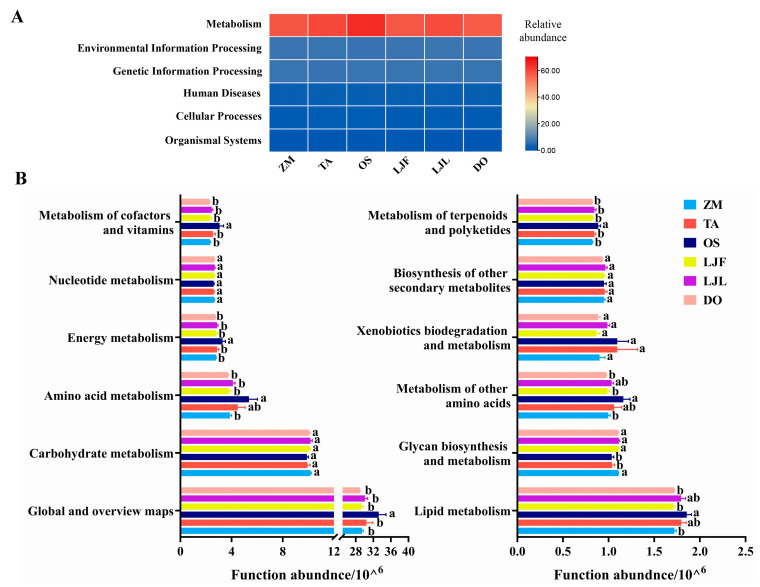
Comparison of KEGG function prediction in the gut bacterial communities of *Spodoptera frugiperda* fed on different host plants. (**A**) heatmap of pathway level one; (**B**) analysis of metabolic pathway level two. Abbreviations in the figure represent different treatments: ZM, maize; TA, wheat; OS, rice; LJF, honeysuckle flowers; LJL, honeysuckle leaves; and DO, Chinese yam. Different lowercase letters above bars indicate significant differences among different treatments (*p* < 0.05, One-way ANOVA).

The PICRUSt2 results showed that the functional prediction categories of the gut bacteria of *S. frugiperda* feeding on different hosts were focused on metabolism, environmental information processing, genetic information processing, human diseases, cell transformation, and organismal systems, and the relative abundance of the metabolic pathway was the highest (Figure 6A). In addition, there were significant differences in the relative abundance of the secondary classification level of metabolic pathways among each treatment (Figure 6B). Specifically, the relative abundance of amino acid metabolism, energy metabolism, cofactors, and vitamins metabolism in the gut bacteria of *S. frugiperda* feeding on rice were significantly higher than those of other treatments (Figure 6B).

## 4. Discussion

Insect gut microbes are important to the expansion of host populations and they enhance the adaptability to external adverse factors [13,15]. Numerous research demonstrated that the structure and diversity of insect gut bacteria can be significantly affected by host diets [15,20,21]. Our study compared the changes of gut bacterial communities of *S. frugiperda* fed on two traditional Chinese herbal medicines and three major food crops. The diversity and abundance of the microbial community of *S. frugiperda* fed on various host plants were significantly different. The abundance and diversity of gut bacterial community of *S. frugiperda* larvae fed on rice were higher than that on other host plants. It was similar to the research that the gut bacterial diversity in *G. molesta* and *P. xylostella* was significantly changed after the host plants shifted [20,21].

At the phylum level, Firmicutes, Actinobacteriota, and Proteobacteria dominated microbial communities of *S. frugiperda* larvae. Among them, Firmicutes was predominant in all samples (relative abundance >80%). This result was similar to the intestinal bacterial community of many Lepidoptera insects, such as *S. exigua*, *S. litura*, and *P. xylostella* [42,43,44]. Lv et al. also reported that Firmicutes were dominant bacterial phyla in gut bacterial communities of *S. frugiperda* reared on different host plants [33]. Numerous studies indicated that Firmicutes bacteria are indispensable in the material and energy metabolism of the host [13,45,46]. Firmicutes can synthesize a variety of carbohydrate-degrading enzymes and convert glycogen into nutrients available to the host. Firmicutes can also produce sugar transporters, especially for mannose, which can reduce the toxicity of hazardous substances for insects [47,48]. Besides, Ptaszyńska et al. found that Firmicutes may participate in the infection of honeybees by pathogenic bacteria [49]. The high relative abundance of Firmicutes in all samples in our study supports their importance in the digestion and metabolism processes of *S. frugiperda*. The high level abundance of Actinobacteriota occurred in *S. frugiperda* larvae fed on wheat and rice. It has been reported that Actinobacteria is involved in various metabolic and physiological activities, including extracellular enzyme production and antimicrobial activity [50].

Enterococcaceae was the most common gut bacteria in Lepidoptera, such as *G. molesta*, *Manduca sexta*, *Spodoptera littoralis*, and *Helicoverpa armigera* [15,51,52,53]. In our study, Enterococcaceae was also a dominant family of microorganisms with the highest abundance in all treatments. Researchers have confirmed that Enterococcaceae play a positive role in host adaptability, which was related to the degradation of the plant cell walls into available nutrients and the metabolization of toxic secondary metabolites [53,54,55]. Recent studies have found that the *S. frugiperda* invading China has a higher fitness level when fed on maize than when fed on rice [19]. He et al. reported that the survival rate of *S. frugiperda* larvae fed on rice was only 0.4%, and that they were unable to pupate successfully [29]. We found that the relative abundances of Enterococcaceae in *S. frugiperda* larvae fed on rice was significantly lower than that on maize. This phenomenon provided a new idea to reveal that the *S. frugiperda* damage to maize was more serious than to rice in China. *Bacillus* and *Enterbacter* were detected in our study with high relative abundance in the *S. frugiperda* fed on honeysuckle leaves. Bacillus is one of the main symbiotic bacteria that produces digestive enzymes in insects, which can produce cellulase, protease, and lipase [56,57]. *Enterbacter* plays an important role in glucose metabolism, which can also degrade cellulose [21,58]. These two genera both had the highest abundance in *S. frugiperda* fed on honeysuckle leaves, which may be related to the high content of cellulose in honeysuckle leaves.

*Klebsiella* within Enterobacteriaceae is a beneficial microbe, which wildly exists in the guts of insects and other herbivores [59,60]. *Klebsiella* could increase the fitness of hosts and promote the development and reproduction of insects [21]. Acevedo et al. found that *Klebsiella* was able to inhibit the defense response of plants, which could improve the survival rate and the environmental adaptability of *S. frugiperda* larvae [61]. Niyazi et al. reported that *Klebsiella* could promote the reproductive capacity of *Ceratitis capitata* [62]. In our study, *Klebsiella* was detected with a high relative abundance in *S. frugiperda* fed on maize, wheat, and honeysuckle leaves than that on other diets. Furthermore, our previous study showed that the *S. frugiperda* in these three groups had shorter larval development duration, higher larval survival rate, and higher reproductive ability than other groups [30]. These results suggested a potential relationship between the abundance of *Klebsiella* and the development and reproduction of *S. frugiperda*.

## 5. Conclusions

In this study, high-throughput sequencing was used to compare the gut bacterial communities of *S. frugiperda* reared on different host plants. The results confirmed that the host plants can significantly affect the gut bacterial diversity and structure of *S. frugiperda*. *Spodoptera frugiperda* fed on rice had higher bacterial richness and diversity. Firmicutes, Actinobacteriota, and Proteobacteria were the most abundant bacterial phyla. PICRUSt2 analysis indicated that most predicted functions were related to metabolism. This investigation of gut microbiota can supply a theoretical basis for clarifying the host adaptation mechanism of *S. frugiperda*.

## Figures and Tables

**Table 1:** The relative abundance of dominant bacteria at the phylum and family taxonomic levels in *Spodoptera frugiperda* fed on different hosts.

Taxonomy	Group	ZM	TA	OS	LJF	LJL	DO	*F*-Value	*p*-Value
Phylum	Firmicutes	98.39 ± 1.21 a	80.80 ± 12.71 b	83.93 ± 2.47 ab	96.68 ± 4.68 a	94.04 ± 0.90 ab	98.97 ± 0.44 a	5.731	0.006
	Actinobacteriota	0.37 ± 0.20 b	13.33 ± 3.89 a	11.90 ± 2.26 a	0.12 ± 0.06 b	0.51 ± 0.16 b	0.25 ± 0.08 b	35.936	<0.001
	Proteobacteria	0.79 ± 0.10 b	0.40 ± 0.15 b	0.75 ± 0.38 b	0.47 ± 0.27 b	5.17 ± 0.87 a	0.69 ± 0.47 b	51.616	<0.001
	Cyanobacteria	0.54 ± 0.31 a	0.02 ± 0.01 b	0.14 ± 0.21 ab	0.02 ± 0.02 b	0.03 ± 0.05 b	0.01 ± 0.01 b	5.565	0.007
	Bacteroidota	0.04 ± 0.03 bc	0.01 ± 0.01 c	0.57 ± 0.13 a	0.01 ± 0.01 bc	0.19 ± 0.07 b	0.07 ± 0.05 bc	34.112	<0.001
Family	Enterococcaceae	92.35 ± 5.94 a	82.25 ± 6.20 ab	68.50 ± 9.75 b	92.97 ± 5.94 a	90.80 ± 0.54 a	97.48 ± 2.07 a	9.537	0.001
	Staphylococcaceae	0.01 ± 0.00 b	0.62 ± 0.44 b	34.31 ± 11.58 a	0.03 ± 0.02 b	0.05 ± 0.01 b	0.01 ± 0.00 b	26.099	<0.001
	Micrococcaceae	0.17 ± 0.16 b	4.62 ± 0.74 a	3.84 ± 2.01 a	0.05 ± 0.02 b	0.01 ± 0.00 b	0.01 ± 0.00 b	18.391	<0.001
	Enterobacteriaceae	0.53 ± 0.23 b	0.22 ± 0.03 bc	0.34 ± 0.08 bc	0.35 ± 0.23 bc	2.38 ± 0.09 a	0.04 ± 0.04 c	110.248	<0.001
	Erysipelatoclostridiaceae	0.09 ± 0.01 b	0.74 ± 0.11 a	0.21 ± 0.06 b	0.01 ± 0.00 b	0.14 ± 0.23 b	0.00 ± 0.00 b	20.716	<0.001

Abbreviations in the table represent different treatments: ZM, maize; TA, wheat; OS, rice; LJF, honeysuckle flowers; LJL, honeysuckle leaves; and DO, Chinese yam. The data are the mean ± SE. The letters indicate the significant differences in relative abundance in the same taxon between different treatment groups (one-way ANOVA, Tukey’s test, α = 0.05).

## Data Availability

The data presented in this study are available in the article.

## Supplementary Materials

The following supporting information can be downloaded at: https://www.mdpi.com/article/10.3390/insects14030264/s1, Figure S1: Alpha diversity index rarefaction curves. (A) Sobs index (B) Shannon index; Table S1: Statistical analysis of V3–V4 hypervariable region of 16S rDNA of the bacteria in the gut of *S. frugiperda* larvae; Table S2: Analysis of alpha-diversity of midgut bacteria in different treatments of *S. frugiperda* larvae.
