# Supplementary material for: Effect of Different Host Plants on the Diversity of Gut Bacterial Communities of Spodoptera frugiperda (J. E. Smith, 1797)
Source: Insects. 2023 Mar 8;14(3):264. doi: 10.3390/insects14030264 (PMC10053068; doi:10.3390/insects14030264)
Supplement: Supplementary file 1 [file insects-14-00264-s001.zip › Supplementary Table S1.pdf]

Table S1. Statistical analysis of V3-V4 hypervariable region of 16S rDNA of the bacteria in the gut of *S. frugiperda* larvae

Sample Info	Seqences number	Base number	Average length	Min-length	Max-length
ZM1	57881	24825179	428.900313	203	442
ZM2	49251	21127748	428.981097	213	527
ZM3	37072	15929264	429.684506	203	431
TA1	55532	23770496	428.050421	277	431
TA2	67027	28470045	424.754875	278	442
TA3	55129	23614478	428.349471	276	465
OS1	52070	22167876	425.732207	247	431
OS2	56596	24163903	426.954255	257	507
OS3	39112	16783452	429.1126	277	431
LJF1	53804	23098274	429.304029	242	431
LJF2	62462	26795789	428.993452	277	443
LJF3	49066	20974198	427.469082	208	517
LJL1	50691	21773840	429.54055	310	433
LJL2	55302	23753009	429.514466	203	490
LJL3	58633	25166187	429.215408	403	438
DO1	50711	21746236	428.826803	239	431
DO2	50647	21720704	428.864572	262	431
DO3	59655	25600103	429.135915	203	443
