# Supplementary material for: Effect of Different Host Plants on the Diversity of Gut Bacterial Communities of Spodoptera frugiperda (J. E. Smith, 1797)
Source: Insects. 2023 Mar 8;14(3):264. doi: 10.3390/insects14030264 (PMC10053068; doi:10.3390/insects14030264)
Supplement: Supplementary file 1 [file insects-14-00264-s001.zip › Supplementary Table S2.pdf]

Table S2 Analysis of Alpha-diversity of midgut bacteria in different treatments of *S. frugiperda* larvae

Sample Name	Sobs	Ace	Chao1	Shannon	Simpson	Coverage
ZM1	100	0.1854	0.9530	135.9146	151.23	0.998966
ZM2	67	0.5316	0.7543	83.5884	77.69	0.999469
ZM3	64	0.3136	0.8741	142.0279	106.27	0.999134
TA1	54	0.5913	0.7870	118.3254	84.00	0.999413
TA2	61	1.3300	0.4498	134.7937	88.14	0.999441
TA3	63	0.7839	0.6848	134.2135	118.20	0.999329
OS1	159	1.9987	0.2300	164.9866	180.00	0.999581
OS2	136	1.4126	0.4076	168.4564	161.11	0.998938
OS3	74	0.9976	0.4945	106.9823	122.33	0.999162
LJF1	24	0.0383	0.9912	34.6864	31.20	0.999749
LJF2	43	0.4784	0.7858	57.1088	51.67	0.999637
LJF3	52	0.4617	0.8339	71.2333	65.33	0.999553
LJL1	99	0.5580	0.8319	109.5521	110.77	0.999497
LJL2	118	0.5724	0.8311	129.0256	127.50	0.999469
LJL3	145	0.6244	0.8140	171.5438	173.33	0.999022
DO1	71	0.0850	0.9808	134.2395	98.35	0.999134
DO2	77	0.3206	0.9026	110.3119	106.08	0.999218
DO3	79	0.1661	0.9572	97.3257	93.88	0.999357
