# Supplementary figures and images for: Effect of Different Host Plants on the Diversity of Gut Bacterial Communities of Spodoptera frugiperda (J. E. Smith, 1797)
Source: Insects. 2023 Mar 8;14(3):264. doi: 10.3390/insects14030264 (PMC10053068; doi:10.3390/insects14030264)

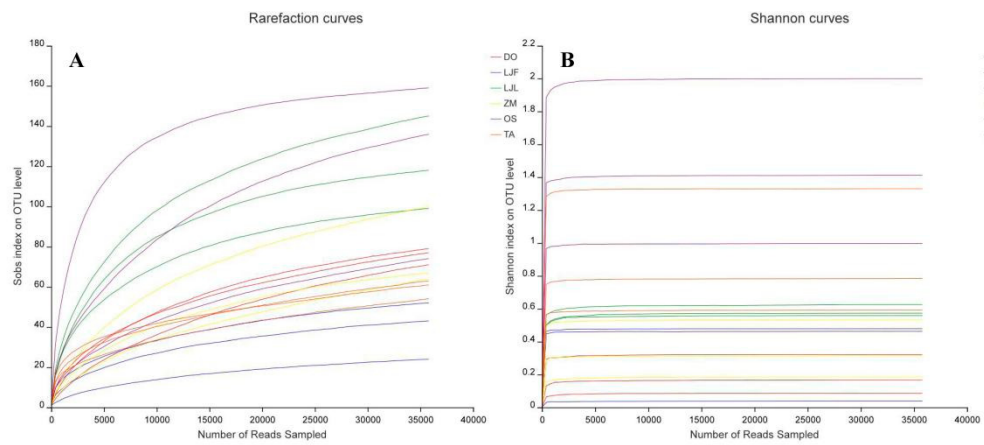

Figure S1. Alpha diversity index rarefaction curves. (A) Sobs index (B) Shannon index

Supplement: Supplementary file 1 [file insects-14-00264-s001.zip › Supplementary Figure S1.pdf]
